# Isolation and Characterization of Nanocellulose from *Polypodiophyta* Fern Using Chemo-Mechanical Method

**DOI:** 10.3390/biomimetics9100624

**Published:** 2024-10-14

**Authors:** Katja Vasić, Monika Dokl, Željko Knez, Maja Leitgeb

**Affiliations:** 1 Faculty of Chemistry and Chemical Engineering, University of Maribor, Smetanova ulica 17, 2000 Maribor, Slovenia; katja.vasic@um.si (K.V.); monika.dokl@um.si (M.D.); zeljko.knez@um.si (Ž.K.); 2 Faculty of Medicine, University of Maribor, Taborska ulica 8, 2000 Maribor, Slovenia

**Keywords:** nanocellulose, isolation, *Polypodiophyta* fern, chemo-mechanical method, biomaterial, biopolymer

## Abstract

Nanocellulose is considered a promising and sustainable biomaterial, with excellent properties of biorenewability with improved mechanical properties. As a unique natural biopolymer, it has been applied to many different industries, where efficient and environmentally friendly productions are in demand. For the first time, ferns from the class *Polypodiopsida* were used for the isolation of cellulose fibers, which was performed using a chemo-mechanical method. As chemical treatment plays a crucial role in the isolation of nanocellulose, it affects the efficiency of the extraction process, as well as the properties of the resulting nanocellulose. Therefore, mechanical fibrillation was performed via grinding, while the chemical process consisted of three different treatments: alkali treatment, bleaching, and acid hydrolysis. In three different experiments, each treatment was separately prolonged to investigate the differing properties of isolated nanocellulose. Structural analysis and morphological analysis were investigated by SEM, EDS, FT-IR, and DLS. The thermal stability of cellulose fibers was investigated by TGA/DSC. The morphology of obtained nanocellulose was confirmed via SEM analysis for all samples, with particles ranging from 20 nm up to 600 nm, while the most consistent sizes were observed for NC3, ranging from 20 to 60 nm. FT-IR spectra showed prominent absorption peaks corresponding to cellulose, as well as the absence of absorption peaks, corresponding to lignin and hemicellulose. The EDS confirmed the elemental purity of nanocellulose, while TGA/DSC indicated higher thermal stability of nanocellulose, compared to untreated fern, which started to degrade earlier than nanocellulose. Such characteristics with unique properties make nanocellulose a versatile biomaterial for the industrial production of cellulosic materials.

## 1. Introduction

Biomaterials from renewable sources are gaining immense attention as environmental sustainability dictates today’s world of science and everyday life. Environmentally friendly approaches are used to try to avoid environmental issues caused by the use of synthetic materials and biopolymers. Therefore, such materials are being replaced by innovative and renewable biomaterials, namely biopolymers. In that manner, agricultural feedstocks are the most abundant alternative, as well as the biggest source for such biomaterials, as they can be used to obtain various high-value-added products with minimum impact on the environment and its ecosystem. Cellulose, being the most abundant biopolymer and the oldest on Earth, plays a significant role in the polymer industry for the synthesis of green-oriented products and was used in the forms of cotton, fiber, or hemp as its first sources in ancient times. Cellulose is a natural biopolymer with many advantages since it displays non-toxicity, inherent biocompatibility, biodegradability, renewability, low cost, and colloidal stability. It can also be derived from many different biomass feedstocks [1,2,3]. However, when starting the process, pretreatment of cellulose is often required, in order to efficiently fractionate cellulose from other lignocellulosic fractions [4]. With the purpose of recovering cellulosic nanosized biomaterials, it is necessary to perform the purification of cellulosic fractions by solubilizing the existing non-cellulosic compounds. As different physiochemical methods can be used for the pretreatment of cellulose, a wide range of structurally diverse nanocellulosic biomaterials can be produced [5,6]. The high-demand interest in nanocellulose (NC) is not only in its distinctive nanoscaled structure but also in its intrinsic properties, which include nanoscaled sizes, large specific surface areas, high aspect ratios, and ease of surface chemistry modifications. As it is derived from sustainable sources, it also possesses many desirable functions, which widen its use in different sustainability-oriented industries, such as biomedical applications, sensors, paper, packaging, coatings, and cosmetics. Based on its biocompatible nature, such NC-oriented biomaterials have widened the range of use in environmental remediation as adsorbents and photocatalysts, as well as flocculants, and show high binding affinities and adsorption capacities. Cellulose is a linear polysaccharide with the formula (C_6_H_11_O_5_)_n_ consisting of many repeating β-D-glucopyranose molecules, where the so-called cellobiose units are a dimer of glucose, which are covalently linked through glycosidic bonds between the hydroxyl groups in the carbon C1 and carbon C4 atoms of glucose units [7]. Elementary fibrils are the basic building block units of cellulose fibers, which are the smallest morphological units that form microfibrils [8].

Naturally occurring cellulose is known as cellulose I, which exists in parallel strands without intersheet hydrogen bonding. Cellulose II is thermodynamically more stable and exists in antiparallel strains with intersheet hydrogen bonding. Both cellulose I and II are the main polymorphs of cellulose, as they exist as a variety of different conformations and are a result of different cellulosic chain orientations of hydrogen bonds within the network [9]. The difference in properties of cellulose I and II arises due to changes in crystal structure. The non-cellulosic components, such as lignin, and hemicellulose, as well as other compounds, are removed by pretreatment. Afterwards, NC is isolated from cellulose fibrils using different isolation methods. For biomass pretreatment, alkaline treatment and acid hydrolysis are used [10,11], also known as the delignification process. After the bleaching process, the white-colored fibers of holocellulose (mainly consisting of hemicellulose and cellulose) indicate that lignin and other impurities were successfully removed. The main isolation methods mainly include acid and enzymatic hydrolysis, as well as mechanical processes. Various types of acids, mostly sulfuric acid, formic acid, hydrochloric acid, stearic acid, and maleic acid, were used for acid hydrolysis of cellulosic biomass [12,13,14]. Sulfuric acid causes strong isolation of nanocrystalline cellulose and esterification of hydroxyl groups by sulfate ions. It removes amorphous regions and increases the crystallinity of the fibers. Treatment success is affected by acid concentration, reaction time, and temperature [15]. Different reaction times (5, 10, 15, 30, and 60 min) with a sulfuric acid concentration of 64% (*w*/*w*) were studied by Prado et al., and after 30 min of reaction, the cellulose-based nanoparticles were isolated [16]. Also, TEMPO-mediated oxidation was investigated for the isolation of cellulose nanofibers, which involves the 2,2,6,6-tetramethylpiperidine-1-oxyl radical [17,18]. Enzymatic hydrolysis may be conducted by a variety of enzymes (commonly named cellulases), which are used to break down the β-(1,4)-glycosidic linkages [19]. Due to an impact on the defibrillation of cellulose fibers, several intensive mechanical treatments may be performed. These processes consist of grinding, high-pressure homogenization, steam explosion, cryocrushing, and high-intensity treatments with ultrasound. A combination of chemical and mechanical treatment was reported in many research studies. For example, deep eutectic solvent pretreatment for lignin-containing nanocellulose production was investigated, which displayed good thermal stability and high crystallinity [20]. A report by Almeida et al. describes the production of functionalized nanocellulose using deep eutectic solvents, which resulted in highly fibrillated and functionalized lignocellulose nanofibers [21]. Additionally, Wang et al. report on a new nanocellulose from waste coconut shell fibers, which was based on an ultrasonic method [22], while Zhang et al. investigated the extraction of nanocellulose from banana pseudo-stem, which was conducted using a chemical–mechanical method [23]. NC was also isolated from palm oil mesocarp fiber with hydrothermal treatment, which was coupled with weak acid hydrolysis, such as oxalic acid dihydrate. Two different concentrations of oxalic acid dihydrate were selected for the acid hydrolysis process, where average particle sizes of NC were measured using TEM and were approx. 25 nm [24]. Isolation of nanocellulose from hardwood pulp was performed via phytic acid pretreatment, which yielded around 90% of nanocellulose with a ribbon-like structure [25]. Another study reports on the formation of NC through the two-step approach of delignification, which was microwave-assisted followed by ultrasonic extraction, which produced cellulose of purity 93% and hemicellulose of purity 99% [26]. Also, NC crystals were prepared via acid hydrolysis from tea stalks in China, which reports improved physical and chemical properties, as well as better stability [27]. NC, being a result of the isolation process from lignocellulosic biomaterials, has many advantages; besides biodegradability and renewability, it also has high specific strength and modulus. NC can be divided into two types, namely cellulose nanofibrils (CNFs), which are traditionally extracted via acid treatment, where the amorphous regions are cleaved, obtaining highly crystalline and rigid nanostructures. Since, during acid hydrolysis, the incorporation of acid results in the surface charge of CNCs, colloidal dispersion is quickly formed [28]. Cellulose nanocrystals (CNCs) are most commonly prepared by a mechanical disintegration process. Additionally, various other methods, such as enzymatic treatment, chemical treatment, high-pressure homogenization, ball milling, and ultrasonic fiber delamination, are other approaches used to obtain CNFs [28,29]. Both CNCs and CNFs are obtained via mechanical treatments, such as homogenization, ultrasonication, steam explosion, nanofibrillation, and biologically or chemically assisted milling [30]. It can also be produced with enzymatic treatment, acid hydrolysis, ultrasonication, or hydrothermal treatment [31]. Fern, from the class *Polypodiopsida*, which is a class of nonflowering vascular plants, is a carbohydrate-based source with essential characteristics of plant-fiber-based nanocellulose, since it possesses stems, true roots, and complex leaves and is produced by spores. Such characteristics of this class are molecular, mechanical, and tensile properties, as well as its biodegradability potential. Since ferns are extremely diverse in form, habitat, and reproductive methods, they are abundant in damp and warm places [32,33]. Consequently, they are a cheap and, most importantly, carbohydrate- and nanocellulose-rich source with outstanding structures and features. With ever-growing interest and a combination of multidisciplinary fields, such as life sciences, chemistry, biology, physics, and engineering, they are an important part in the evolution of natural and nano-based biomaterials [34], which are used for constructing tissue replacements or in tissue regeneration and repair, as implants, as well as in drug delivery and biosensing [35,36].

There are a lot of possible applications for such biomaterials. As far as we know, there is no information on isolating NC from ferns; therefore, in the present study, *Polypodiopsida* ferns were used as a raw plant biomaterial for the first time and NC was successfully isolated via a combination of mechanical and chemical treatment. Each chemical treatment (alkali, bleaching, and acid hydrolysis) was prolonged in three different experiments, to investigate the differing properties of resulting nanocellulose. After chemo-mechanical treatment, isolated NC was further characterized, where morphology, chemical structure, and composition were investigated. This work introduces a potential material that may be used in dye extraction, the removal of ion pollution, and the monitoring of emerging pollutants, as well as in drug delivery systems and neuroendovascular treatments.

## 2. Materials and Methods

### 2.1. Raw Materials

Ferns (class *Polypodiopsida*) were collected from a forest in Maribor, Slovenia. Sodium hydroxide (NaOH, microgranular, pure) was obtained from Poch, Poland. Hydrogen peroxide (H_2_O_2_, 30%, p.a.) was obtained from Merck, Germany. Hydrochloric acid (HCl, 37%) and sulfuric acid (H_2_SO_4_, 95–97%, p.a.) were obtained from Honeywell, Fluka, Charlotte, NC, USA.

### 2.2. Isolation of Nanocellulose

**Figure 1:**
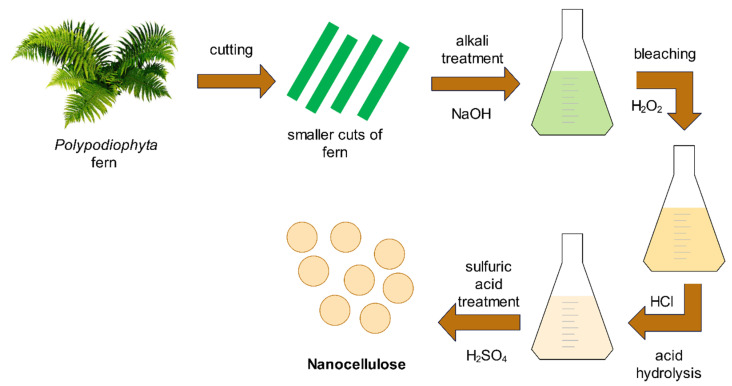
Isolation process of nanocellulose from *Polypodiophyta* fern.

The isolation of nanocellulose is schematically presented in Figure 1. Cellulose was isolated in three different ways. The experimental setup for each sample is presented in Table 1. For all experiments, a small amount of untreated raw material (150 g) was first ground into small pieces using a commercial miller at room temperature and 20,000 rpm. Later, 100 g of grounded material was treated with a 2% aqueous NaOH solution heated to 80 °C for 4 or 24 h under continuous stirring.

The alkali-treated material was then bleached in a 5% H_2_O_2_ aqueous solution at 80 °C for 4 or 24 h under continuous stirring. The bleached material then went through acid hydrolysis with a 2% aqueous HCl solution at 80 °C for 4 or 24 h under continuous stirring. The distilled water/material suspension was put in an ice bath and treated with 60% concentrated H_2_SO_4_, which was added dropwise. The suspension was heated to 50 °C for 1 h, while continuously stirred.

After each treatment, suspensions were filtered and washed with distilled water. Since the solution was highly acidic after the treatment, the material was washed with distilled water until the solution reached neutral pH 7. Obtained fibers were eventually dried for 24 h at 60 °C. The fiber-to-solution ratio used in all treatments was 1:10 (in g/mL). The reaction time during H_2_SO_4_ treatment was the same in each experiment. The reaction time of alkali treatment, bleaching, and acid hydrolysis varied. All experiments were performed in triplicate with a standard deviation of less than ±3%.

### 2.3. Isolation of Nanocellulose

#### 2.3.1. Scanning Electron Microscope (SEM)

The morphology and surface of the isolated samples was analyzed by scanning electron microscopy (SEM) and energy-dispersive X-ray spectroscopy (EDS). The samples were covered with gold coating and performed on a Scanning Electron Microscope (FE, SEM SIRIN, 400 NC, FEI).

#### 2.3.2. Energy-Dispersive X-ray Spectroscopy (EDS)

The elemental composition of the cellulose samples was measured by energy-dispersive X-ray spectroscopy (EDS). Measurements of samples covered with gold coating were performed on a Scanning Electron Microscope (FE, SEM SIRIN, 400 NC, FEI).

#### 2.3.3. Fourier-Transform Infrared (FTIR) Spectroscopy

The functional groups in fibers were analyzed by FTIR analysis and determined in a Perkin Elmer 1600 spectrometer by taking the spectrum in the range of 500 to 4000 cm^−1^. The samples were ground into powder and blended with KBr followed by pressing the mixture into thin pellets.

#### 2.3.4. Thermogravimetric Analysis (TGA)

The thermal stability of the cellulose samples was analyzed with a TGA/DSC 1 analyzer, STARe System, Mettler Toledo, Greifensee, Switzerland at a heating rate of 10 °C/min, ranging from 30 °C to 600 °C. All measurements were performed under nitrogen flow to prevent thermo-oxidative degradation.

#### 2.3.5. Differential Scanning Calorimetry (DSC)

Using a TGA/DSC 1 analyzer, STARe System, Mettler Toledo, the thermal behavior of the cellulose sample was analyzed at a heating rate of 10 °C/min, ranging from 30 °C to 600 °C under a nitrogen atmosphere.

#### 2.3.6. Dynamic Light Scattering (DLS)

The particle size of the isolated samples was determined by Dynamic Light Scattering using a particle size analyzer from the Malvern Zetasizer Nano series. Analyses of aqueous suspensions were carried out for 50 s in the following conditions: water refractive index 1.330, viscosity 0.8872 mPas, and temperature 25 °C.

## 3. Results

Isolation of NC derived from various sources underwent different treatments as per the literature. However, the isolated materials did not possess the same crystal characteristics, as there is a wide range of cellulosic feedstocks, such as plants, animals, bacteria, fungi, or marine algae being used for NC isolation. There are also different (pre)treatments used to obtain such NC. In our study, isolation of NC was performed using *Polypodiopsida* fern for the first time, where it was successfully achieved by three modified treatments, based on different experimental conditions. Different parts of the treatment were varied. As prolonging the time of each chemical treatment could result in different characteristics of isolated nanocellulose, each treatment was carried out for 24 h in three different experiments. In the first experiment (NC1), the alkali treatment was performed for 24 h, which was followed by 4 h of bleaching and 4 h of acid hydrolysis. In the second experiment (NC2), the acid hydrolysis treatment was performed for 24 h, to which the sample was modified beforehand with 4 h of alkali treatment and 4 h of bleaching. In the third experiment (NC3), the alkali treatment was performed for 4 h, which was followed by 24 h of bleaching treatment, and treatment continued for 4 h with acid treatment. All three kinds of treatments were followed by 1 h of sulfuric acid treatment, while alkali bleaching and acid treatment were concluded. Details about each experimental condition and for how long each treatment lasted are presented in Table 1. After the treatments were concluded, the characterization of all obtained samples was examined to investigate how each treatment affected the morphological and structural properties of the samples, as well as the size of the obtained nanoparticles and their distribution. Thus, characterization was examined by performing SEM, EDS, FT-IR, TGA, DSC, and DLS analysis.

### 3.1. Microscopic Morphology Analysis by SEM

**Figure 2:**
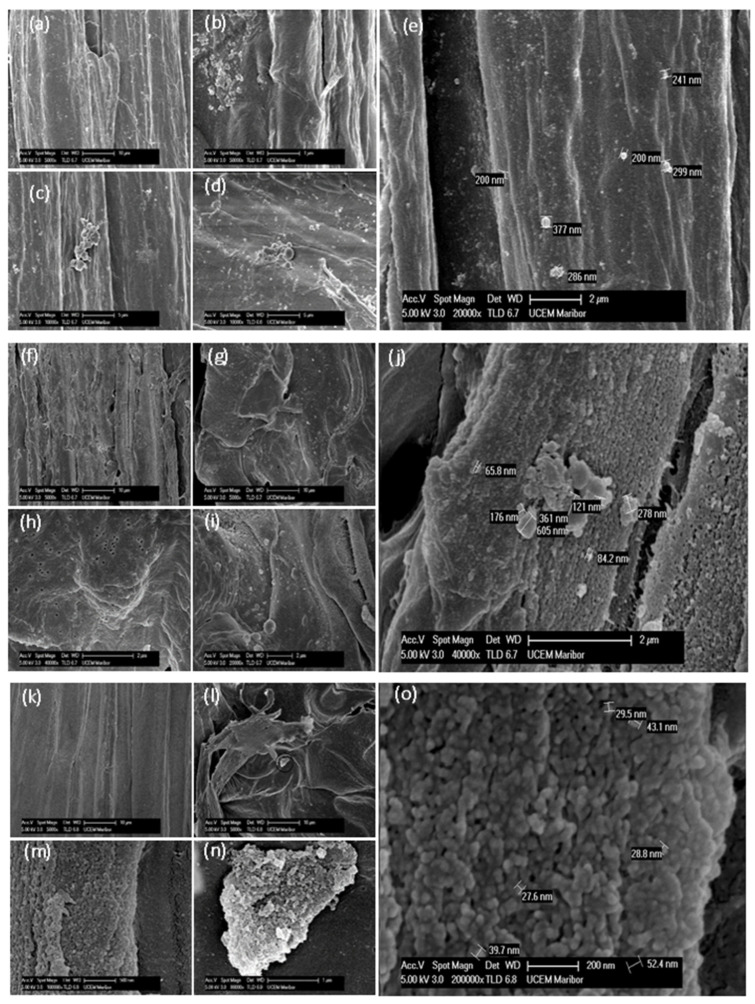
SEM of samples NC1 (**a**–**e**), NC2 (**f**–**j**), and NC3 (**k**–**o**) at various magnification levels.

The surface of the isolated fibers was studied by SEM. Depending on the treatment conditions, fibers and particles of different shapes attached to them were found. The particle size was determined at higher magnification levels. The sample obtained under prolonged alkali treatment contained straight and unbroken fibers measuring approximately 30 µm wide (Figure 2a). According to Figure 2c,e, two clustered particles measuring approximately 5 µm in length are shown. The particles were also arranged in layers (Figure 2b) or dispersed over fibers (Figure 2e). The diameters of the individual particles and the particles grouped together were between 100 and 400 nm. The straight, torn, twisted, and porous fibers were found in SEM images of sample NC2 (Figure 2f–j). The straight fibers were 10 to 20 µm wide. The porosity of fiber as the holes with diameter within the range of 50–100 nm was observed (Figure 2h), indicating the absence of particles that size. The 60 to 600 nm large particles were located on the fiber surface (Figure 2j) with a lightly brushed surface. The prolonged bleaching treatment resulted in straight and twisted fibers (Figure 2k,o). At higher magnification levels, large layers and clusters of small particles ranging from 27 to 60 nm were determined (Figure 2k–o).

It was already investigated and reported in the literature that nanocellulose and other nanocellulosic structures, such as cellulose nanocrystals or cellulose nanofibrils, consist of chains with different order degrees, which vary from arrangements that are highly crystalline to slightly unsettled chain distributions [37]. For sample NC1 (Figure 2a–e), the size varies around 200 and 300 nm, while sample NC2 (Figure 2f–j) displays crystal sizes from 65 to 86 nm, as well as around 600 nm. For sample NC3, the crystal sizes decreased and ranged from 27 to 43 nm (Figure 2k–o). With each treatment, the nanocellulose is distinct and clear. Notably, the NC3 sample exhibits smaller and more even sizes, while NC1 and NC2 exhibit more uneven sizes, which is attributed to the irregular distribution of lignin. NC2 and NC3 demonstrate more uneven sizes, indicating a high aspect ratio. This characteristic can be valuable in composite engineering applications, where such ratios are desirable.

### 3.2. EDS Elemental Analysis

**Figure 3:**
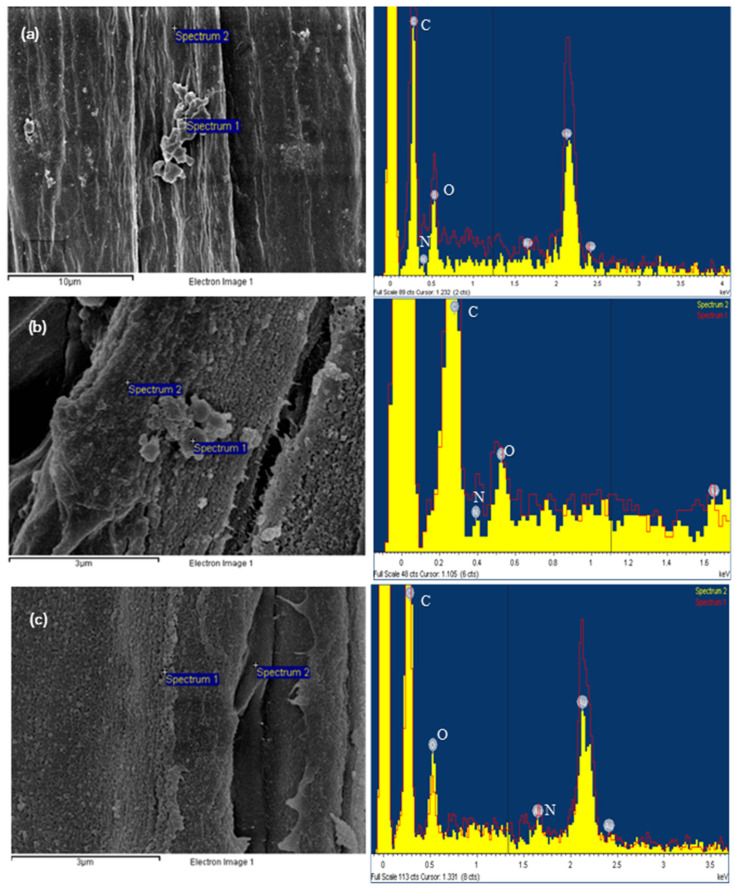
Scanning electron micrographs and line scanning EDS profiles of isolated cellulose samples: (**a**) NC1, (**b**) NC2, and (**c**) NC3.

The insight into the chemical composition of nanocellulose samples was provided by EDS analysis. On the SEM images, two measurement locations of each sample were determined, one on the particle, and the other on the remaining fiber. In the EDS profiles in Figure 3, well-defined peaks characteristic of carbon, oxygen, and nitrogen due to gold coatings were found. For all samples, a high carbon content and a lower oxygen content were revealed. The difference in peak expressivity was large because of dehydration [38]. The difference in nitrogen content of particle and fiber was observed in all samples and might be due to the different shapes of the parts being measured. Because measurements were performed at a low voltage (5 kV), some elements such as sulfur could not be detected. They may be present in the amorphous regions in the samples by reason of higher exposure of hydroxyl groups to esterification [39].

### 3.3. FT-IR Analysis

**Figure 4:**
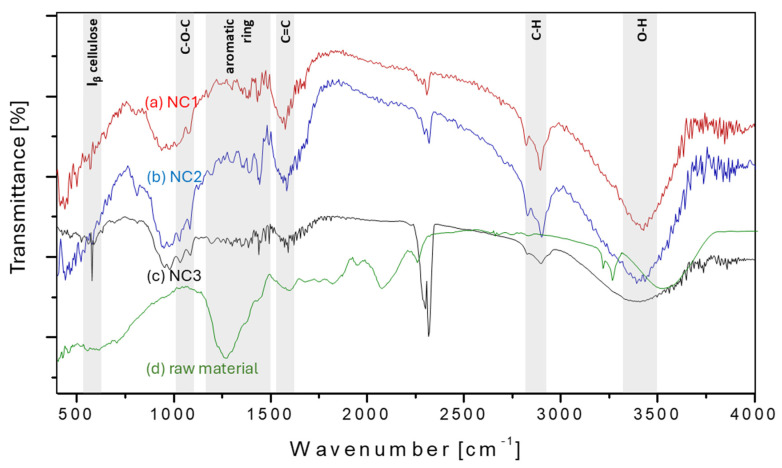
FTIR spectra of the isolated nanocellulose samples: (**a**) NC1, (**b**) NC2, (**c**) NC3, and (**d**) raw untreated material.

FT-IR spectroscopy has been extensively used in research regarding cellulose and NC research, to study the functional groups and to obtain information on chemical changes occurring during various chemical and mechanical treatments. The three main components found in samples are cellulose, hemicellulose, and lignin, which are mainly composed of alkanes, aromatic, esters, ketones, and alcohols [40]. Determined functional groups in the structure of the samples NC1, NC2, NC3, and untreated raw material by infrared transmittance spectra are shown in Figure 4. A broad peak at the range from 3400 to 3440 cm^−1^ was observed in all samples (Figure 4a–c), which can be associated with free O-H stretching vibrations of -OH groups bending due to the presence of inter- and intramolecular hydrogen bonds, which allow chain linkage and structure stability [41]. Consequently, due to the formation of hydrogen bonds via hydroxyl groups during drying, small particles were united into crystalline structures, which increased the surface tension of the fibers. The vibration and elongation of C-H bonds in cellulose and hemicellulose was observed in all three samples, which was pronounced at 2922 cm^−1^, while in Figure 4c, the peak was less pronounced, but still indicates the removal of hemicellulose, as in the other two samples (Figure 4a,b). All presented samples’ characteristic peaks around 1510 cm^−1^ corresponded to the vibration of aromatic C=C in-plane symmetrical stretching vibration of the aromatic ring in lignin [42]. However, all bands assigned to lignin were not observed in the NC1–NC3 samples, which may be successfully removed through the chemo-mechanical process. The absorption bands at approximately 1300–1500 cm^−1^ correspond to the aromatic ring in lignin; hence, the presence of lignin was confirmed for raw material, which was successfully removed in all NC1–NC3 samples. In Figure 4c, a smaller peak was observed, indicating a successful removal of the lignin. Also, the observation of the less pronounced peak at 1647 cm^−1^ correlated to the lower content of absorbed water in cellulose compared to the other two samples [43]. A group of three peaks in the range from 1010 to 1165 cm^−1^ was observed in all samples, suggesting that the groups -CH, -CH_2_, and -OH formed a C-O-C glycosidic ether band and caused vibrations of the skeletal pyranose ring [44]. In Figure 4c, the pronounced peak at 668 cm^−1^ occurred due to the presence of a β-glycosidic bond, and thereby, I_β_ cellulose was determined [45]. FT-IR analysis successfully identified functional groups that were present in each sample and indicated typical band patterns for cellulose, hemicellulose, and lignin, and that were present in all three samples. However, all of the above characteristics indicated that each sample maintained its original structural characteristics. Nevertheless, it was evident that there was a difference in the bandwidth of each spectrum around 2900 cm^−1^, 1600 cm^−1^, and 660 cm^−1^. It can be observed that with an increase in the intensity of bleaching treatment using alkaline hydrogen peroxide, sample NC3 had a wider adsorption peak at the mentioned wavenumber positions, as compared to the other two samples, NC1 and NC2. All these characteristics proved that each treatment that occurred had an impact on treated cellulose, where it attacked corresponding functional groups.

### 3.4. Thermogravimetric Analysis

**Figure 5:**
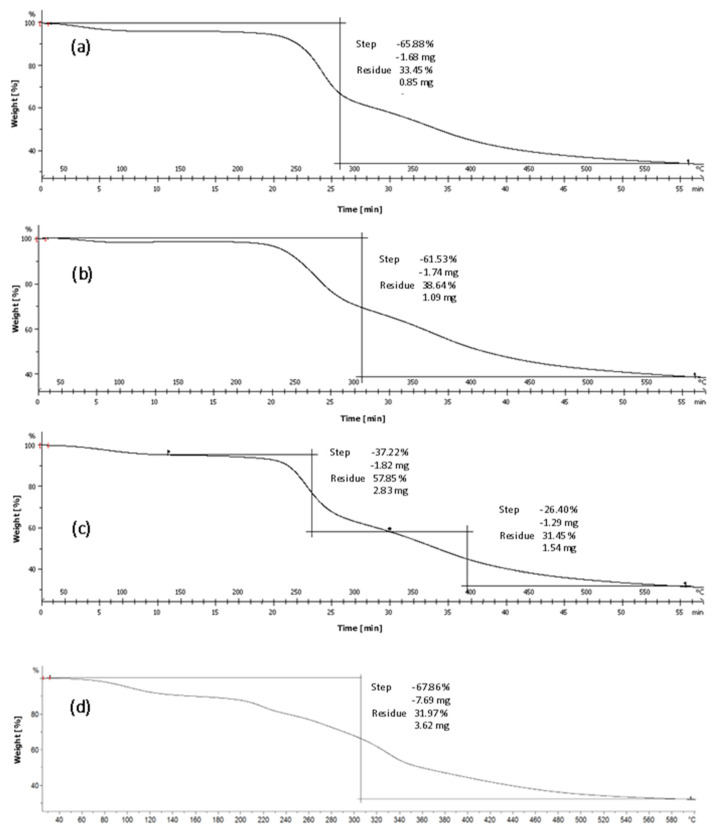
TGA profiles of the isolated cellulose samples: (**a**) NC1, (**b**) NC2, (**c**) NC3, and (**d**) untreated raw material.

As one of the most valuable and important characteristics of NC, the thermal stability of the obtained NC was investigated by thermogravimetric analysis (TGA). Thermal stability presents an important feature of NC, as it is intended as a reinforcing agent in various biomimetic composites. The thermogravimetric curves of the samples (Figure 5) represent weight losses at certain temperatures, and the contents of the samples were determined.

All TGA curves showed thermal degradation of untreated material (Figure 5d) and treated nanocellulose (NC1–NC3) in the N_2_ atmosphere. Generally, the thermostability of the isolated nanocellulose decreases with an increase in pretreatment intensity. For example, compared with untreated cellulose, the maximum decomposition temperature of the NC1, NC2, and NC3 gradually decreased from 340 °C to 285 °C, 305 °C, and 280 °C, respectively. Thermal decomposition of cellulose is divided into three main stages. The initial weight loss was observed in the first stage in the temperature range of 35 °C to 120 °C, which was due to the physically adsorbed water and light volatiles, which were observed in many reports [46,47]. Water evaporates when it is chemically absorbed or loosely bound by intermolecular hydrogen bonds on the surfaces of cellulose. Water can bind to the cellulose structure where sulfate amorphous sites are located and where hemicellulose and lignin have been removed. Since prolonged alkaline treatment was performed, a greater loss of water could be observed in the first sample (Figure 5a), as has also been confirmed in the literature, since this treatment leaves the cellulose structure more open [44]. The second degradation step began at about 230 °C when the sample mass was lost through dehydration, decarboxylation, and decarbonylation. Thermal depolymerization of hemicellulose occurred [48], lasting up to about 320 °C for all samples. Hemicellulose consists of various saccharide groups, such as mannose, xylose, galactose, and glucose in an amorphous structure, which is full of branches that can easily be removed from the main stem at low temperatures [49]. Cellulose pyrolysis begins at higher temperatures because it consists of a long polymer of glucose, which is without branches but has a stronger structure and high thermal stability. In the 230 °C to 280 °C temperature range, crystalline areas began to decompose. With flatter curves in this area for the second sample (Figure 5b), a smaller proportion of crystalline areas were determined. The prolonged acid treatment confirms that this kind of treatment increased the amount of amorphous sites. Hydrocarbon residues and monomeric glucopyranose units break up into free radicals up to about 420 °C [45]. Lignin is decomposed at high temperatures and is considered to be the most thermally stable of the organic components. Because of its complex structure with numerous ether linkages and hydroxyl and methoxy groups, the decomposition of lignin was carried out over a wide temperature range. Decomposition began with the evaporation, melting, and degradation of polysaccharide residues, resulting in loosely bound functional groups. The lignin degradation was in accordance with previously published works [46,47,50]. In the second part, the bigger mass loss occurred, due to the cleavage of the glycosidic bonds and depolymerization of xylan units [40,49]. At 600 °C, 33.45%, 38.64%, and 31.45% of the initial mass of NC1 (Figure 5a), NC2 (Figure 5b), and NC3 (Figure 5c) remained in the samples, respectively. The residue was represented by inorganic substances [51] and non-combustible sulfate esters [45]. Also, a reduction in polymerization degree and particle size was observed in all samples, particularly in sample NC3 (Figure 5c), which was confirmed with TGA. It can be observed from the TGA graphs that the onset of degradation temperature was seen to shift to a higher temperature, indicating that the thermal stability has improved. For NC1, the first step occurs before reaching 300 °C, while for NC2, the first step is visible after 300 °C. Two-stage degradation can be observed in sample NC3, which contributes to the evaporation of water and polysaccharide residues. As an alternative to other alkaline treatments, oxidative alkaline treatments, which use hydrogen peroxide, have reported synergy with many pretreatment methods, such as acetic acid organosolv treatment [52], steam explosion [53], and alkaline extraction [54]. Because of its mild treatment conditions, as well as its low impact on the environment, hydrogen peroxide has become a popular treatment method for biomass fractionation [55].

### 3.5. Differential Scanning Calorimetry (DSC) Analysis

**Figure 6:**
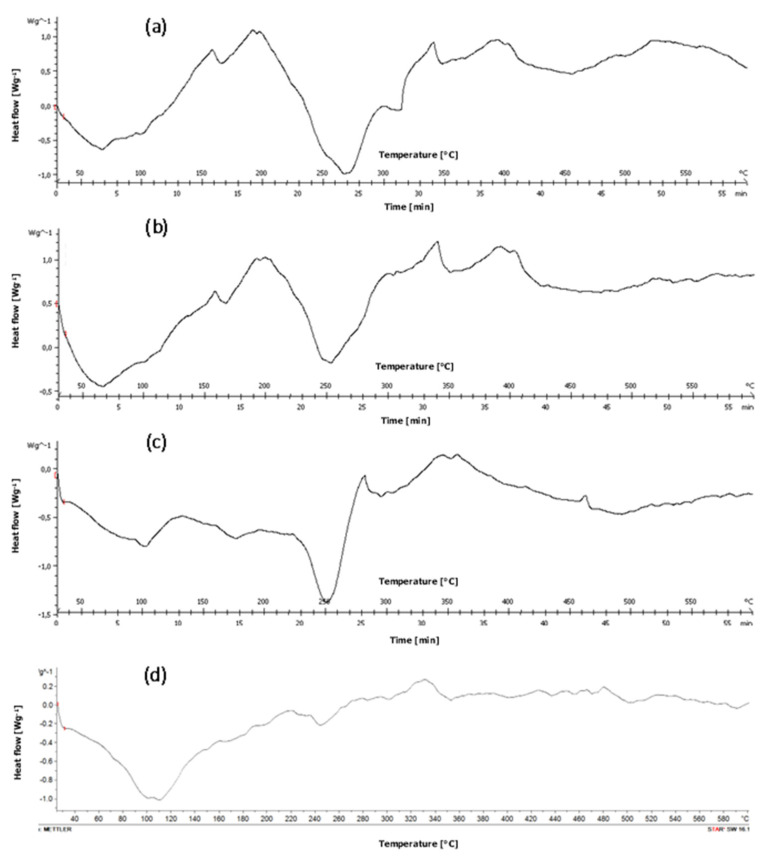
DSC profile curves of the isolated cellulose samples: (**a**) NC1, (**b**) NC2, (**c**) NC3, and (**d**) untreated raw material.

With DSC analysis, additional insights into the thermal stability of the samples were provided. The thermal degradation of the samples as shown with TGA and in Figure 6 occurred in three steps. Firstly, decomposition occurred with the removal of unbound and bound water molecules. The curves have, in addition to the main one, several smaller peaks due to other components that differentially bound and retained water in the structure [44]. The weight loss was distributed over the entire temperature range throughout the diagram because the sample consisted of cellulose, lignin, hemicellulose, pectin, and other components having different melting temperatures. All three DSC thermograms of the samples exhibited distinct endothermic changes occurring within the temperature range since the nature of endotherms is quite characteristic of the composition of the material. The initial endotherm could be observed in all three samples, which occurred at much lower temperatures than 100 °C and happened due to evaporation, causing moisture loss. Such moisture loss occurred also due to sulfuric acid treatment since the sulfuric acid acted as a dehydrating catalyst. Moreover, cellulose crystals were sulfonated on the active surface, which reduced the affinity for moisture absorption. Therefore, some moisture could be adsorbed on the surface; however, it was loosely bound and could evaporate at a much lower temperature. The main degradation step included dehydration reactions and the formation of volatile products. Most cellulose and hemicellulose decomposed in the range of 200–290 °C, which is evident from Figure 6d, and most of the decomposition for raw material occurred at 100 °C. The third degradation step for samples NC1–NC3 occurred above 400 °C with a long swing of the curve corresponding to the decay of lignin. Degradation of the first sample took place at a higher temperature than the other two samples, which proved higher thermal stability. As a result of the alkaline treatment, its structure was more orderly. After the removal of hemicellulose and lignin, the hydrolysate could diffuse into the fibers more easily. Treatment with sulfuric acid involved the esterification of hydroxyl groups, and sulfates acted as catalysts for the hydrolysis of pyranose bonds, which linked cellulose units to longer chains. Owing to the size and abundance of the sulfate groups, the cellulose structure was rearranged and the amount of amorphous regions increased, which led to an extensive reduction in the polymerization degree and, consequently, particle size [44].

### 3.6. Particle Size Distribution Studies

**Figure 7:**
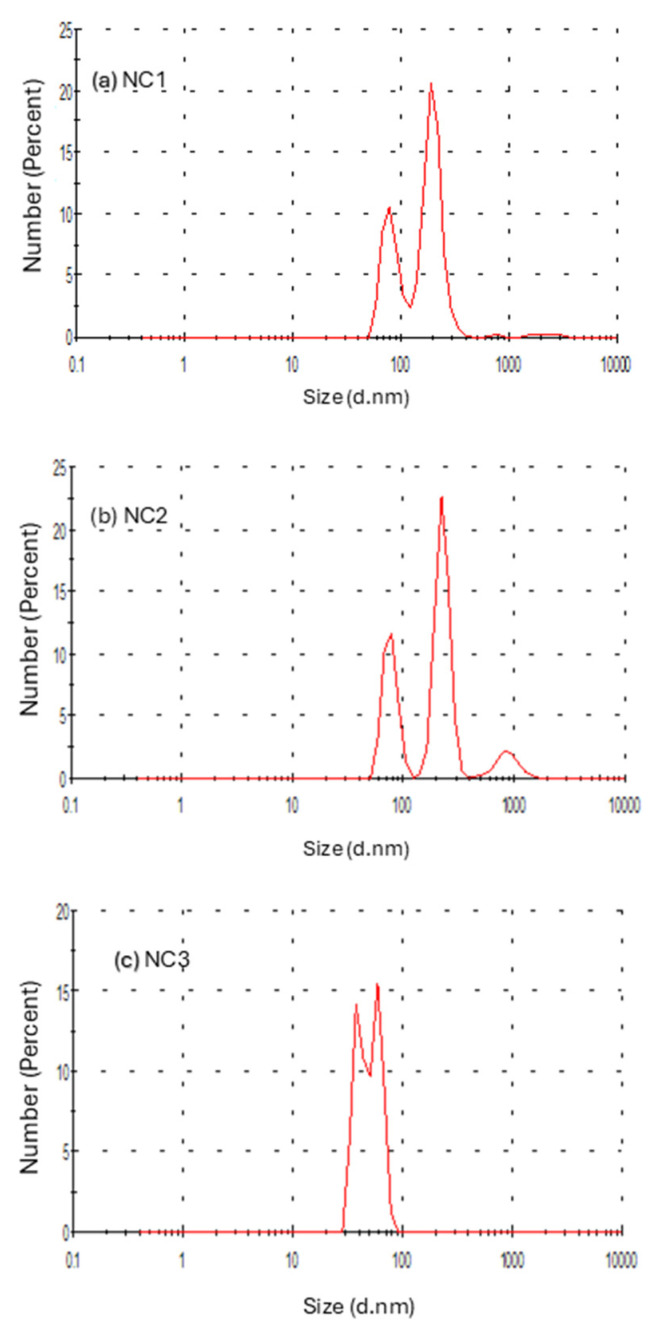
Particle size distribution profiles of isolated cellulose samples.

The DLS technique is commonly used to obtain and verify particle sizes and their stability, but also to report on the statistical distribution of the particles present in NC. Obtained measurements considered spherical particles, and the values depended on the orientation of the fibers in suspension; in most cases, they were higher than those measured by microscopic analysis. According to Figure 7a, suspension NC1 showed two peaks with mean values around 200 nm and 83 nm, with intensities of 64.7% and 33.8%, respectively. For suspension NC2 (Figure 7b), a distribution in three different ranges was observed, where mean values were near 76 nm, with an intensity of 32.2%, and a more significant population (59.3%) with average sizes of 227 nm and 877 nm and an intensity of 8.5%. For suspension NC3 (Figure 7c), the measured particles were within the 50–100 nm range.

The most consistent particle sizes were observed in sample NC3, where bleaching treatment was prolonged. Alkaline bleaching using hydrogen peroxide is essentially a delignification process, which can result in the solubilization of a considerable amount of hemicellulose since it induces depolymerization and cleavage of ester bonds cross-linking lignin and hemicellulose [56]. The DLS analysis is in accordance with results obtained from SEM and TGA, which confirms that the most consistent sizes are obtained when the cellulose is treated with prolonged bleaching treatment using alkaline hydrogen peroxide.

## 4. Conclusions

In the present study, nanocellulose was isolated from *Polypodiopsida* fern under three different prolonged treatment conditions. Each chemical process consisted of alkaline and acid treatment, as well as bleaching and sulfuric acid treatment. In three different experiments, alkaline, bleaching, and acid hydrolysis treatments were prolonged to 24 h. The prolonged alkaline treatment resulted in a more orderly structure of nanocellulose in sample NC1, while the prolonged acid treatment confirmed more amorphous sites on the surface of nanocellulose in sample NC3. FT-IR analysis confirmed that each prolonged treatment had an impact on the isolated nanocellulose in all samples of NC1, NC2, and NC3. It is evident from our study that varying the time of each chemical process by prolonging its time of treatment can affect the end characteristics of isolated nanocellulose and in doing so, enhance its quality and potential for use in various industrial applications. Such new NC biomaterials may be used for industrial coatings and suspensions, as well as stabilizing agents for water-based paints. Such advanced product development and its optimization are crucial in adding value to derived nanocellulose, which is necessary in the development of advanced and feasible nanocellulose-based biopolymer materials. Additionally, to overcome increasing environmental and economic concerns, milder reaction conditions and easier recyclability are needed.

## Figures and Tables

**Table 1:** Experimental conditions for each sample.

Experiment	Alkali Treatment (NaOH)	Bleaching (H_2_O_2_)	Acid Hydrolysis (HCl)	Sulfuric Acid Treatment (H_2_SO_4_)
NC1	24 h	4 h	4 h	1 h
NC2	4 h	4 h	24 h	1 h
NC3	4 h	24 h	4 h	1 h

## Data Availability

All data generated or analyzed during this study are included in this published article.
